# Common Variable Immunodeficiency and Neurodevelopmental Delay Due to a 13Mb Deletion on Chromosome 4 Including the NFKB1 Gene: A Case Report

**DOI:** 10.3389/fimmu.2022.897975

**Published:** 2022-06-17

**Authors:** Clara Franco-Jarava, Irene Valenzuela, Jacques G. Riviere, Marina Garcia-Prat, Mónica Martínez-Gallo, Romina Dieli-Crimi, Neus Castells, Laura Batlle-Masó, Pere Soler-Palacin, Roger Colobran

**Affiliations:** ^1^Immunology Division, Vall d’Hebron University Hospital, Universitat Autònoma de Barcelona (UAB), Barcelona, Spain; ^2^Translational Immunology Research Group, Vall d’Hebron Research Institute (VHIR), Vall d’Hebron University Hospital, Barcelona, Spain; ^3^Jeffrey Modell Diagnostic and Research Center for Primary Immunodeficiencies, Barcelona, Spain; ^4^Department of Clinical and Molecular Genetics, Vall d’Hebron University Hospital, Barcelona, Spain; ^5^Medicine Genetics Research Group, Vall d’Hebron Research Institute (VHIR), Vall d’Hebron University Hospital, Barcelona, Spain; ^6^Infection in Immunocompromised Pediatric Patients Research Group, Vall d’Hebron Research Institute (VHIR), Vall d’Hebron University Hospital, Barcelona, Spain; ^7^Pediatric Infectious Diseases and Immunodeficiencies Unit, Vall d’Hebron University Hospital, Barcelona, Spain

**Keywords:** primary immunodeficiencies, syndromic immunodeficiencies, common variable immunodeficiency, nfkb1, chromosomal rearrangements

## Abstract

Syndromic immunodeficiencies are a heterogeneous group of inborn errors of immunity that can affect the development of non-immune organs and systems. The genetic basis of these immunodeficiencies is highly diverse, ranging from monogenic defects to large chromosomal aberrations. Antibody deficiency is the most prevalent immunological abnormality in patients with syndromic immunodeficiencies caused by chromosomal rearrangements, and usually manifests as a common variable immunodeficiency (CVID)-like phenotype. Here we describe a patient with a complex phenotype, including neurodevelopmental delay, dysmorphic features, malformations, and CVID (hypogammaglobulinemia, reduced pre-switch and switch memory B cells, and impaired vaccine response). Microarray-based comparative genomic hybridization (aCGH) revealed a 13-Mb deletion on chromosome 4q22.2-q24 involving 53 genes, some of which were related to the developmental manifestations in our patient. Although initially none of the affected genes could be linked to his CVID phenotype, subsequent reanalysis identified *NFKB1* haploinsufficiency as the cause. This study underscores the value of periodic reanalysis of unsolved genetic studies performed with high-throughput technologies (eg, next-generation sequencing and aCGH). This is important because of the ongoing incorporation of new data establishing the relationship between genes and diseases. In the present case, *NFKB1* had not been associated with human disease at the time aCGH was performed. Eight years later, reanalysis of the genes included in the chromosome 4 deletion enabled us to identify *NFKB1* haploinsufficiency as the genetic cause of our patient’s CVID. In the future, other genes included in the deletion may be linked to human disease, allowing us to better define the molecular basis of our patient’s complex clinical phenotype.

## Introduction

Inborn errors of immunity (IEI) are a large heterogeneous group of disorders affecting various components of the immune system. To date, more than 450 genes causing inborn errors of immunity have been identified, involving several molecular disease mechanisms (gain of function, loss of function, dominant negative, haploinsufficiency) and modes of inheritance (X-linked, autosomal dominant, and recessive) (1, 2). The clinical phenotype of primary immunodeficiencies can include immune-related conditions (greatly varying, but related to susceptibility to infection, autoimmunity, inflammation, allergy, immune dysregulation, or malignancy), or clinical conditions that are not directly related to immune system dysfunction and affect other organ systems. These latter conditions are collectively termed syndromic immunodeficiencies. The genetic bases of syndromic immunodeficiencies vary considerably, but they can be classified into two major categories. First, monogenic defects affecting single genes that are usually pleiotropic, meaning they control or have an effect on two or more seemingly unrelated phenotypic traits. The International Union of Immunological Societies (IUIS) Expert Committee of Inborn Errors of Immunity lists the conditions caused by these genes under the category: combined immunodeficiencies with syndromic features. In the last IUIS update, the category included 63 genes causing 58 different disorders (1). And second, chromosomal rearrangements involving different genes. Here, the spectrum of genetic defects is huge, encompassing aneuploidies such as Down syndrome (trisomy 21) and Turner syndrome (monosomy X), both known to be associated with immunodeficiency (3, 4), as well as more restricted defects such as 22q11 microdeletions (5). In addition to these well-known conditions, other private and sporadic (ie, affecting only a single patient) chromosomal rearrangements involving immunodeficiency have been described. In 2016 we participated in a European Society for Immunodeficiencies (ESID) working group that published a retrospective, observational survey study describing 46 patients with chromosomal aberrations associated with immunodeficiency (largest reported cohort to date) (6). In some cases the specific gene/s causing the immune phenotype can be identified (7), but in many others, they remain unknown (6). According to the ESID survey published by Schatorjé et al, antibody deficiency is the most prevalent immunological abnormality in syndromic immunodeficiency caused by chromosomal rearrangements, usually manifesting as a common variable immunodeficiency (CVID)-like phenotype (6).

CVID, the most common primary immunodeficiency, occurring in approximately 1:25,000 individuals, is an exclusion diagnosis based on clinical and immunological criteria. It is a clinically and genetically heterogeneous disorder characterized by susceptibility to infection, a poor vaccine response, hypogammaglobulinemia, and immune dysregulation (8, 9). Despite the increasing number of studies using next-generation sequencing tools, only 10% to 20% of CVID cases have a known underlying genetic cause (10, 11). In Europeans, pathogenic variants of the nuclear factor κB subunit 1 (*NFKB1*) gene are the most common monogenic cause of CVID, accounting for 4% of cases in a cohort of 390 unrelated CVID cases (12). More than 100 different *NFKB1* variants have been described in CVID patients, mainly single nucleotide variants (SNVs) or small deletions/insertions (indels) causing a variety of deleterious effects on the protein (13).

Here, we report the clinical and molecular findings of a patient with a 13-Mb deletion in chromosome 4, comprising 53 genes. The patient has a complex phenotype, including neurodevelopmental delay, dysmorphic features, malformations, and immunodeficiency. Although initially none of the affected genes could be linked to his CVID phenotype, subsequent reanalysis allowed us to identify *NFKB1* haploinsufficiency as the cause of these manifestations.

## Case Description

The proband is a 25-year-old male, the only child of non-consanguineous Caucasian parents. Both parents were healthy and had no family history of congenital abnormalities, intellectual disability, or immune disorders. Fetal ultrasound in the third trimester of gestation detected a unilateral dysplastic kidney and oligohydramnios. A prenatal diagnosis was not performed. Delivery was induced at 38 weeks of gestation: birth weight was 2410g (-1.95SD), length 45cm (-2.75SD), and occipital-facial circumference 32cm (-1.38SD). At birth, he was noted to have facial dysmorphism with hypertelorism, a broad nasal tip and low-set ears, and a unilateral preauricular skin tag. Bilateral single palmar crease, 2-3 toe syndactyly, cryptorchidism, and hypospadias were also noted. Axial hypotonia was evident since birth. Abdominal ultrasound confirmed the unilateral dysplastic kidney. Echocardiography and transfontanellar ultrasound findings were normal. The electroencephalogram and ophthalmological examination were normal.

**Figure 1:**
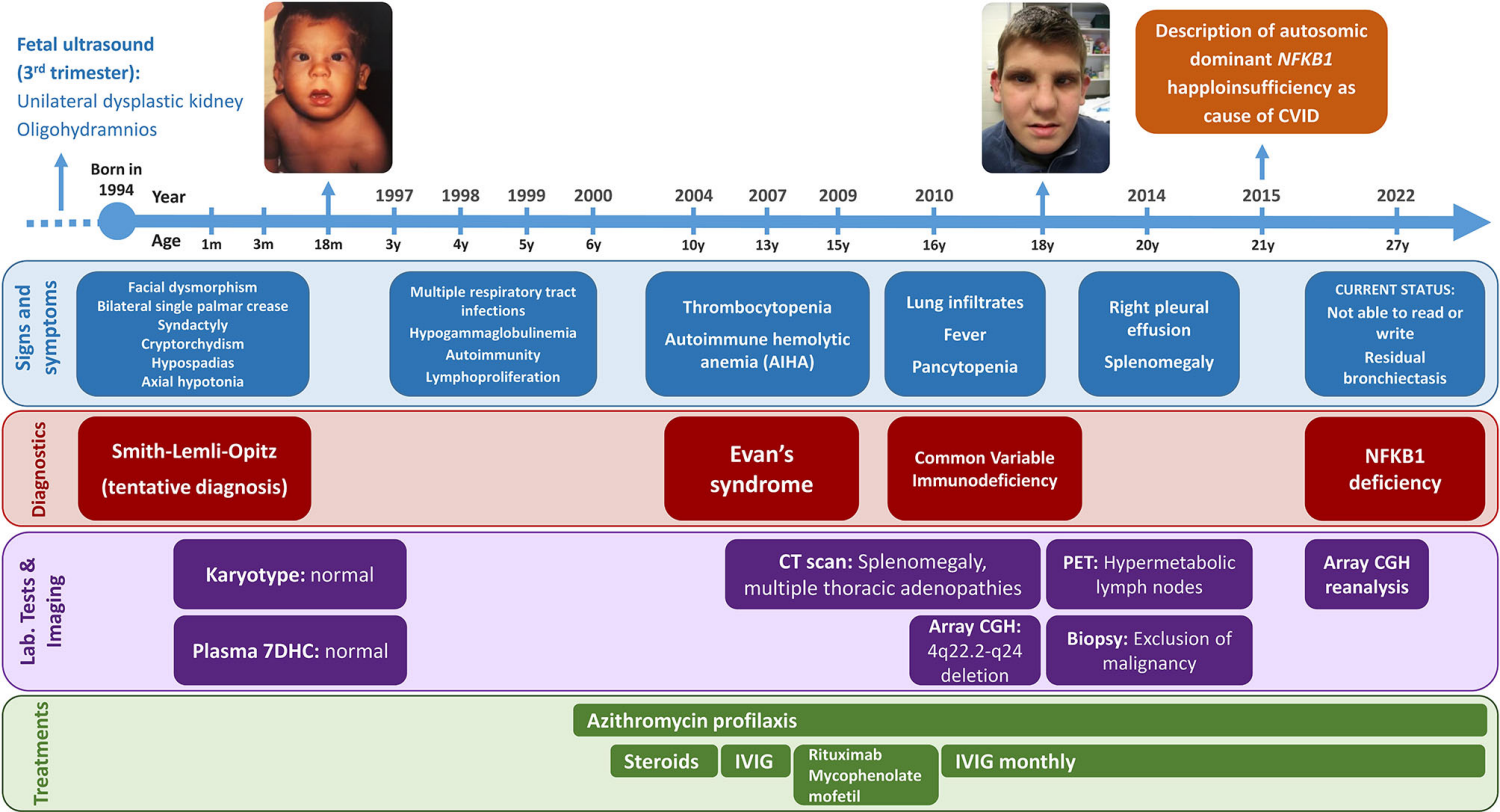
Timeline of the patient’s clinical history. Items are classified into signs and symptoms (blue), diagnoses (red), laboratory tests and imaging (purple), and treatments (green). The patient’s images are included with the explicit consent of the patient and his parents.

The patient was first admitted to our hospital within one month of life due to failure to thrive. Hypogammaglobulinemia and hypogonadism were detected at that time. He was seen in our Clinical Genetics Department at 3 months of age and was noted to have a narrow forehead, horizontal eyebrows, synophrys, deep-set eyes, epicanthus, wide nasal ridge, anteverted nares with underdeveloped alae nasi, and a thin upper lip vermilion with smooth philtrum (**Figure 1**). Karyotype testing and plasma 7-dehydrocholesterol (7DHC) analysis, based on the initial suspicion of Smith-Lemli-Opitz syndrome, were normal. Progressive developmental assessment showed a notable delay, as he walked independently at the age of 3, had poor fine motor control, and poor expressive language. At the age of 25 years, he remains unable to read or write.

From the immunological perspective, the patient showed marked immune system dysregulation early in life, with infections, autoimmunity and lymphoproliferation. Between 3 months and 6 years of age, he was repeatedly hospitalized for recurrent upper and lower respiratory tract infections caused by respiratory viruses and encapsulated bacteria (including various episodes of pneumonia), which led to residual bronchiectasis. At that point, prophylactic treatment with azithromycin was started. Later, the patient presented with Evans syndrome. Initially, at 10 years of age, he experienced only thrombocytopenia, which had a good response to steroids. At the age of 13 years, he showed autoimmune hemolytic anemia together with thrombocytopenia and received intravenous immunoglobulin therapy. Two years later, because of recurrent thrombocytopenia episodes, the patient received rituximab and mycophenolate mofetil, with a good clinical response. At the age of 16 years, he was hospitalized because of lung infiltrates, fever, and pancytopenia. Computed tomography study revealed splenomegaly and multiple thoracic lymph nodes. After ruling out ALPS-related disorders, a diagnosis of CVID was established based on the patient’s autoimmune cytopenia, sustained hypogammaglobulinemia, and absent isohemagglutinins; immunoglobulin replacement therapy was started. He also showed an impaired response to *Haemophilus influenzae* type b (Hib) conjugate vaccine and tetanus toxoid vaccine, and a normal response to pneumococcal polysaccharide vaccine. At the age of 20 years, the pulmonary condition progressed to right pleural effusion, and positron emission tomography detected hypermetabolic lymph nodes on both sides of the diaphragm, including increased spleen uptake. Malignancy and clonal rearrangements were excluded by lymph node biopsy (**Figure 1**).

## Extended Immunological Characterization of the Patient

At the age of 16 years when the patient was diagnosed with CVID, he showed hypogammaglobulinemia with low IgG and IgM, and absent IgA. Absolute levels of T and B cell lymphocyte subpopulations were low, despite normal percentages (**Table 1**). T cell immunophenotyping revealed that the decreased total numbers of CD4 T cells are mostly due to the decrease in naive CD4 T cells. Absolute numbers of CD8 T cells were also reduced. Distribution of CD4 T helper subpopulations showed a Th1-skewed profile. B cell immunophenotyping revealed a maturation stop at naïve subpopulations. Most B cells were naïve, and there was a marked reduction in pre-switch and switch memory B cells (**Table 1**).

**Table 1:** Immunological findings in a patient with a 13-Mb deletion (including the *NFKB1* gene) in chromosome 4q22.2-q24.

Immunological parameters	At CVID diagnosis (16 years old)*	Reference Values
**Immunoglobulins (mg/dL)**		
**IgG**	↓ **225**	700 - 1600
**IgM**	↓ **52**	70 - 400
**IgA**	↓ **<10**	40 - 230
**IgE (KU/L)**	**<2**	0 - 117
**IgD**	↓ **<13**	13 - 152
**IgG subclasses (mg/dL)**		
**IgG1**	↓ **170**	261 - 1081
**IgG2**	↓ **40**	112 - 408
**IgG3**	96	22 - 288
**IgG4**	5	4 - 86
**Lymphocyte subpopulations 10^9^/L (%)**		
**T cells (CD3+)**	↓ **0.49** (66)	1.1 - 2.6 (62–81)
**T helper (CD3+CD4+)**	↓ **0.29** (39)	0.6 - 1.5 (31–53)
**T cytotoxic (CD3+CD8+)**	↓ **0.15** (21)	0.3 - 1.0 (19-30)
**B cells (CD19+)**	↓ **0.08** (10)	0.14 - 0.6 (6-21)
**NK cells (CD16+CD56+)**	0.17 (22)	0.15 - 0.7 (6-23)
**Advanced T cell immunophenotyping (%)**		
**Maturation status**		
**CD4 effector memory (CD45RA-CCR7-)**	↑ **72**	23 - 40
**CD4 central memory (CD45RA-CCR7+)**	14	14 - 27
**CD4 TEMRA (CD45RA+CCR7-)**	4.5	4 - 12
**CD4 naive (CD45RA+CCR7+)**	↓ **9.5**	30 - 50
**CD8 effector memory (CD45RA-CCR7-)**	31	22 - 41
**CD8 central memory (CD45RA-CCR7+)**	↑ **9**	0.7 - 5
**CD8 TEMRA (CD45RA+CCR7-)**	↓ **10**	14 - 38
**CD8 naive (CD45RA+CCR7+)**	50	27 - 50
**Differentiation status**		
**Th1 (CD3+CD4+CXCR3+CCR6-)**	↑ **45**	17 - 29
**Th2 (CD3+CD4+CXCR3-CCR6-)**	↓ **34**	48 - 66
**Th1-Th17 (CD3+CD4+CXCR3+CCR6+)**	12	7 - 17
**Th17 (CD3+CD4+CCR6+CXCR3-)**	5	5 - 12
**T regs (CD3+CD4+CD25+CD127-CCR4+CD45RO+)**	0.8	0.8 - 4.3
**Advanced B cell immunophenotyping**		
**Naive (CD19+ IgD+CD27-)**	↑ **87**	48 - 72
**Transitionals (CD19+ IgD+CD27-CD24hCD38h)**	9	2 - 11
**Preswitch memory (CD19+ IgD+CD27+)**	↓ **5**	>13
**Switch memory(CD19+ IgD+CD27+)**	↓ **1.4**	10 - 22

*See case description and **Figure 1** for previous and concomitant medications at the time of the study.

Arrows indicate values that are above or below the reference values.

## Identification of a Large Deletion in Chromosome 4 Affecting 53 Genes

**Figure 2:**
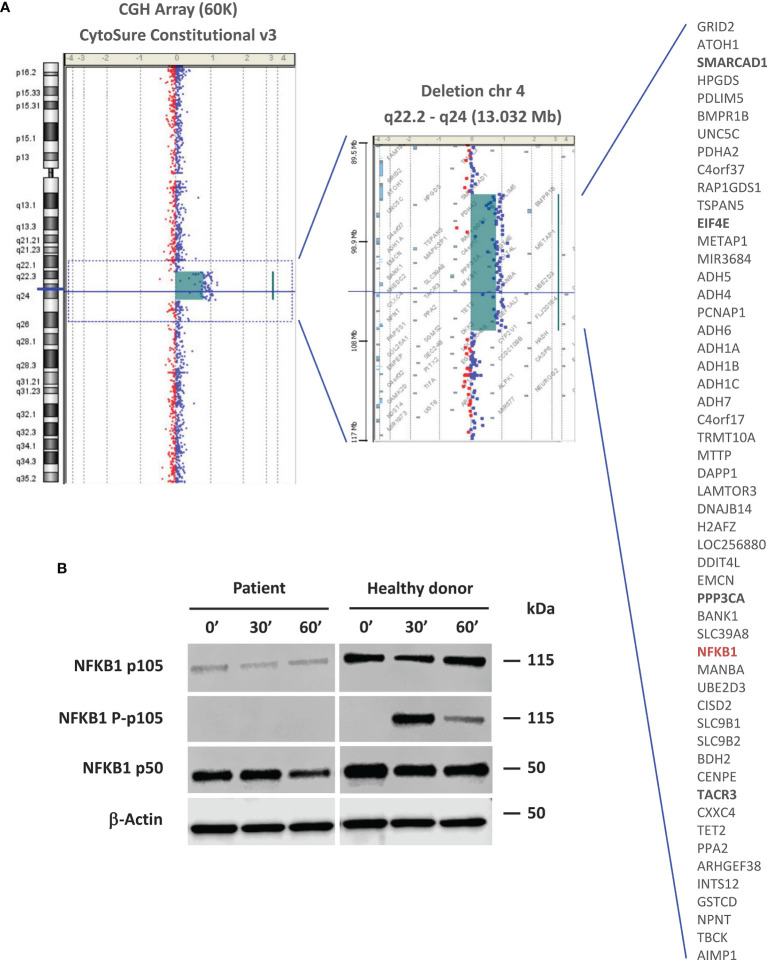
Molecular and functional studies. **(A)** Array CGH-based identification of the 13-Mb deletion in the 4q22.2-q24 chromosomal region. Patient DNA was labeled with cyanine 3 (Cy3, red dots) and control DNA with cyanine 5 (Cy5, blue dots). The image is reported as the ratio of Cy5 and Cy3 fluorescence intensity. The list of genes encompassed by this deletion is shown. Genes that are likely related to the patient’s non-immune clinical phenotype are in bold. The *NFKB1* gene is highlighted in red. **(B)** NF-kB pathway evaluation by western blot. NFKB1 p105/p50 expression and activation were evaluated in PBMC-derived protein extracts from the patient and a healthy donor. For a better comparison between the healthy donor and patient, we quantified the p105 and p50 bands using the ImageJ software. The quantification reflects the relative amounts as a ratio of each protein band relative to the lane’s loading control (β-actin): p105 patient (0’: 0.24, 30’: 0.20, 60’: 0.22); p105 healthy donor (0’: 0.79, 30’: 0.76, 60’: 1.10). p50 patient (0’: 1.07, 30’: 1.12, 60’: 0.74); p50 healthy donor (0’: 1.39, 30’: 1.27, 60’: 1.41).

Because of the patient’s complex phenotype involving immunological abnormalities, dysmorphic features and developmental changes, microarray-based comparative genomic hybridization (aCGH) was performed in 2010 (**Supplementary Methods**), which identified a 13.03-Mb heterozygous deletion on chromosome 4q22.2-q24 (ISCN formula to date: arr [GRCh37] 4q22.2 q24 (94264597_107296595)x1) (**Figure 2A**). This large deletion involved 53 genes and was confirmed to be a *de novo* event by FISH analysis of both parents using a locus-specific BAC probe. A few genes in the 4q22.2-q24 deletion are reported to cause genetic disorders by haploinsufficiency and these could relate to the patient’s developmental manifestations: *SMARCAD1* (Huriez syndrome, OMIM #181600), *EIF4E* (susceptibility to autism, OMIM #615091), and *PPP3CA* (developmental and epileptic encephalopathy, OMIM #617711). No previously described CVID-related gene defect was detected at that time and additional genetic studies were not performed.

## aCGH Reanalysis Revealed *NFKB1* Haploinsufficiency Underlying the CVID Phenotype

In light of the continuous accumulation of new data on the relationship between disease and genes/variants, we usually perform a periodic data reanalysis (every 2-5 years) in cases of unsolved genetic studies using high-throughput technologies (eg, next-generation sequencing or aCGH). In one of these reanalyses performed in 2018, we discovered that one of the genes included in the 13-Mb deletion was *NFKB1* (**Figure 2A**). In 2015, the group led by Bodo Grimbacher reported for the first time monoallelic pathogenic mutations in *NFKB1* as a cause of CVID by a mechanism of haploinsufficiency (14). As the patient’s clinical, laboratory and molecular findings concurred, we considered that *NFKB1* haploinsufficiency was the cause of his CVID phenotype. To exclude pathogenic (P) or likely pathogenic (LP) variants in other genes associated with CVID we performed whole exome sequencing. We did not find any P or LP variants in IEI causing genes based on the IUIS classification (1, 2), including all reported CVID-related genes. Recently, autosomal recessive *TET2* deficiency has been described causing an immune dysregulation syndrome including susceptibility to infection, lymphadenopathy, hepatosplenomegaly, developmental delay, autoimmunity, and lymphoma of B-cell origin (15). Since *TET2* was one of the 53 genes included in the 13-Mb deletion, we analyzed WES data confirming that the other *TET2* allele was present and showed the wild type sequence (without rare genetic variants).

*NFKB1* encodes for a full-length precursor, p105 protein, which gives rise to the p50 active subunit implicated in the canonical NF-κB pathway (16). The effect of NFKB1 haploinsufficiency on p105/p50 expression and activation was evaluated by western blot (WB) of PBMC-derived protein extracts (**Supplementary Methods**), which showed detectable but markedly decreased p105 and p50 levels in the patient compared to those of a healthy donor. p105 phosphorylation was not detected following PMA and ionomycin stimulation (**Figure 2B**).

## Discussion

Syndromic immunodeficiencies caused by private and sporadic chromosomal rearrangements are highly variable and the specific cause of the immunological defect sometimes remains unknown (6). Here, we describe a patient with a complex phenotype including neurodevelopmental delay and immunodeficiency. aCGH disclosed a large (13-Mb) deletion in chromosome 4q22.2-q24 involving 53 genes, which included *NFKB1* as the cause of his CVID phenotype.

Interstitial deletions in the 4q21-q25 region have been reported in a small number of patients so far, and precise mapping of the breakpoints is described in only a few cases (17–21). Four of these patients had a deletion that considerably overlapped the one described in this study (molecular and clinical details summarized in **Table 2**). On analysis of the data from all these cases, we found that the most common clinical features were developmental delay/intellectual disability (100%), hypotonia (100%), congenital heart defects (60%), short stature (60%), and other non-specific dysmorphic features. Interestingly, the two patients with the most concordant deletions (Terada et al. and the current patient) both showed intrauterine growth retardation and genital malformation. Immune system abnormalities have not been described in any patients with interstitial deletions in the 4q21-q25 region. However, in only one case the deletion reported included the *NFKB1* gene (patient 2 in Hilhorst-Hofstee et al.) and the patient’s latest examination took place at the age of 12 years. Two main features of *NFKB1* deficiency are incomplete clinical penetrance (70%) and a highly variable age of onset. According to the largest published study, the mean age at which the first characteristic clinical manifestations occur (mainly infections, autoimmune manifestations, and inflammatory symptoms) is 17 years (ranging from birth to >60 years) (13). Therefore, the patient in the Hilhorst-Hofstee study was likely asymptomatic at the time the paper was written, but he may show symptoms later.

**Table 2:** Clinical and molecular features of reported patients with chromosomal deletions overlapping the 4q22.2-q24 region.

	Terada et al, 2001 (17)	Jacquemont et al, 2006 (21)	Hilhorst-Hofstee et al, 2009 (patient 1) (20)	Hilhorst-Hofstee et al, 2009 (patient 2) (20)	Current study
**Chromosomal 4 deletion** **(coordinates, GRCh38)**	4q21.22-q23 (n.a.)	4q21.23-q23 (n.a.)	4q22.1-q23 (4:88773982-100821184)	4q23-q25 (n.a.)	4q22.2q24 (4: 93343447-106375438)
**Deletion size**	n.a.	17.3 Mb	12 Mb	8.1-9.7 Mb	13.03 Mb
**Inheritance**		*De novo*	*De novo*	*De novo*	*De novo*
***NFKB1* gene included?**	No	No	No	Yes	Yes
**Age at last examination**	4 months	11 years	3 years	12 years	25 years
**Sex**	Male	Male	Male	Male	Male
**Prenatal manifestations**	Intrauterine growth retardation	-	-	-	Intrauterine growth retardation
**Growth retardation**	Yes	-	-	-	Yes
**Dysmorphism**	Frontal bossing, microretrognathia, preaxial polydactyly of the right foot	Two hair whorls, bilateral ptosis, microstomia	Hypotelorism, broad based nose, epicanthus, abnormally shaped head with prominent forehead, short adducted thumbs	Sparse hair, broad nasal tip, unilateral ear tag, narrow forehead	Facial dysmorphism, bilateral single palmar crease, syndactyly
**Hypotonia**	Yes	Yes	Yes	Yes	Yes
**Global developmental delay**	Yes	Yes	Yes	Yes	Yes
**Intellectual disability**	-	Yes	Yes	Yes	Yes
**Short stature**	Yes	-	Yes	-	Yes
**Congenital heart defect**	Yes (VSD, DAP)	Yes (ASD)	Yes (ASD, VSD, DAP)	-	-
**Feeding difficulties**	-	-	Yes (NG tube)	-	-
**Genital anomalies**	Micropenis, hypospadias	-	-	-	Micropenis, hypospadias
**Immunodeficiency**	-	-	-	-	Yes
**Other manifestations**	Epilepsy (3 months) Hepatoblastoma	Congenital hip dislocation Scoliosis	Cleft palate, Pierre Robin sequence	Sleeping problems and constipation.	Unilateral dysplastic kidney

n.a., not available; the symbol “-” means No; VSD, ventricular septal defect; DAP, ductus arteriosus persistens; ASD, atrial septal defect; NG, nasogastric.

We extended our search of chromosomal deletions including *NFKB1* to the Decipher database (https://www.deciphergenomics.org/) and found 11 patients with a range of deletions (from 900 Kb to 14.5 Mb) including *NFKB1* (**Supplementary Table 1**). The age at their last clinical assessment ranged from 1 to 19 years. Interestingly, in one patient the available phenotypic information included decreased circulating antibody levels, concordant with *NFKB1* deficiency.

To date, more than 100 heterozygous *NFKB1*variants have been reported in patients with a CVID-like phenotype (13, 22). Most pathogenic variants are predicted to cause loss of function, but only a few missense variants are truly pathogenic. In a large and comprehensive functional study, Li et al. found that only 57% of published *NFKB1* variants were deleterious, whereas 43% were neutral (22). These authors also demonstrated that deleterious heterozygous *NFKB1* variants cause disease by haploinsufficiency. Therefore, any new *NFKB1* variants, especially missense variants, should be tested with a proper functional assay to determine the functional significance. Conversely, CVID patients carrying rare *NFKB1* missense variants should not be diagnosed as having *NFKB1* deficiency without a functional demonstration of the variant’s pathogenicity (22). Our patient is the first reported case of *NFKB1* haploinsufficiency by complete absence of one *NFKB1* allele, obviating the need for functional studies. However, we showed a marked reduction in p105 and p50 levels by WB. We could not detect p105 phosphorylation after PMA and ionomycin stimulation, probably due to a lack of sensitivity of our WB. A markedly reduced, but detectable, p105 phosphorylation is expected in individuals with *NFKB1* haploinsufficiency.

Two additional patients harboring large *NFKB1* deletions have been described (12). The deletion in the first patient extended from upstream the *NFKB1* gene to intron 17 (del 103370996-103528207). The second patient’s deletion started at *NFKB1* intron 1 and ran downstream of 3’UTR (del 103436974-103652655). Although neither of these deletions involve the whole *NFKB1* gene, they are large enough to imply a de facto haploinsufficiency. In both reported patients and the patient described here the deletion was a *de novo* event and the first clinical signs of *NFKB1* deficiency started during childhood.

*NFKB1* deficient patients present considerable clinical and immunological heterogeneity but most (up to 90%) have hypogammaglobulinemia and this is the reason for the high incidence of bacterial infections, especially those affecting the upper and lower respiratory tract (13). Our patient presented with transient hypogammaglobulinemia at early infancy. Once the patient received rituximab, low B cells and hypogammaglobulinemia persisted thus making difficult to establish an appropriate diagnosis of CVID. It is well described that rituximab may cause long term immunological effects undistinguishable from CVID in susceptible individuals specially with low IgG and IgA levels before therapy (23, 24). Therefore, the role of *NFKB1* as either causative or predisposing gene in this patient remains to be clarified. However, immunologically significant *NFKB1* haploinsufficiency seems to reinforce the major role of this genetic defect in sight of the patient’s clinical and immunological features. Regarding T cell compartment, the Th1-skewed profile found in our patient has been previously described in *NFKB1* deficient patients and associated with inflammatory symptoms (13, 25).

It is important to emphasize that we found the molecular defect associated with the patient’s CVID phenotype by reanalysis of aCGH data. There is now consensus that reanalysis of existing genomic data in unsolved cases increases the diagnostic yield (26, 27). Although systematic reanalysis is usually done with next-generation sequencing data (mainly whole exome or whole genome sequencing), data from other high-throughput technologies can also provide benefits. In the case reported here the large deletion found encompasses 53 genes, most of them still not associated with human disease. This was the case of *NFKB1* at the time in which aCGH was performed in 2010. Eight years later, reanalysis of the genes included in the chromosome 4 deletion led us to define *NFKB1* haploinsufficiency as the genetic defect causing or contributing to the patient’s CVID. In the future other genes included in the deletion may be linked to human disease, allowing us to better define the molecular basis of our patient’s complex clinical phenotype.

## Data Availability Statement

The original contributions presented in the study are included in the article/**Supplementary Material**. Further inquiries can be directed to the corresponding authors.

## Ethics Statement

The studies involving human participants were reviewed and approved by Clinical Research Ethics Committee (CEIC), Vall d’Hebron Research Institute (VHIR). Written informed consent to participate in this study was provided by the participants’ legal guardian/next of kin. Written informed consent was obtained from the individual(s), and minor(s)’ legal guardian/next of kin, for the publication of any potentially identifiable images or data included in this article.

## Author Contributions

CF-J and MG-P designed, performed and analyzed the functional assays (western blot). IV, JR and PS-P provided patient care, and collected and provided clinical data. MM-G supervised and analyzed flow cytometry data. RC and LB-M supervised and analyzed sequencing data. NC performed and analyzed the comparative genome hybridization array. CF-J, IV and RC wrote the manuscript. PS-P and RC designed and supervised the project, provided resources and edited the manuscript. All co-authors reviewed, commented on, and approved the final version of the manuscript. All authors contributed to the article and approved the submitted version.

## Funding

This study was funded by Instituto de Salud Carlos III, grants PI17/00660 and PI20/00761, cofinanced by the European Regional Development Fund (ERDF). This study was also funded by the Jeffrey Modell Foundation. This work is supported by the European Reference Network for Rare Immunodeficiency, Autoinflammatory and Autoimmune Diseases Network (ERN-RITA).

## Conflict of Interest

The authors declare that the research was conducted in the absence of any commercial or financial relationships that could be construed as a potential conflict of interest.

## Publisher’s Note

All claims expressed in this article are solely those of the authors and do not necessarily represent those of their affiliated organizations, or those of the publisher, the editors and the reviewers. Any product that may be evaluated in this article, or claim that may be made by its manufacturer, is not guaranteed or endorsed by the publisher.
