# Supplementary material for: Common Variable Immunodeficiency and Neurodevelopmental Delay Due to a 13Mb Deletion on Chromosome 4 Including the NFKB1 Gene: A Case Report
Source: Front Immunol. 2022 Jun 17;13:897975. doi: 10.3389/fimmu.2022.897975 (PMC9247144; doi:10.3389/fimmu.2022.897975)
Supplement: Supplementary file 1 [file DataSheet_1.pdf]

**Supplementary Table 1**. Individuals with deletions in chromosme 4 including the *NFKB1* gene reported in Decipher database (https://www.deciphergenomics.org/).

| Patient ID | Sex | Chr 4 deletion (coordinates, GRCh38) | Size | Inheritance / Genotype | Phenotype (s) | Age at last clinical assessment |
|---|---|---|---|---|---|---|
| 1580 | male | 4:98747288-106803182 | 8.06 Mb | Unknown / Heterozygous | Abnormal hair morphology, autism, blepharophimosis, broad nasal tip, coarse facial features, facial asymmetry, flat occiput, infra-orbital crease, intellectual disability, sleep disturbance, uplifted earlobe | 8 years |
| 249234 | female | 4:100010584-104308722 | 4.30 Mb | De novo / Heterozygous | Coarse facial features, intellectual disability, macrotia, Scoliosis, Seizure | 10 years |
| 253148 | male | 4:98109249-103106128 | 5.00 Mb | De novo / Heterozygous | n.a. | 16 years |
| 263047 | female | 4:99253457-103085561 | 3.83 Mb | Unknown / Heterozygous | Autism, intellectual disability | 16 years |
| 269925 | male | 4:97840809-108269099 | 10.43 Mb | De novo / Heterozygous | n.a. | 1 year |
| 303816 | female | 4:98416654-103719753 | 5.30 Mb | Unknown / Heterozygous | Abnormal facial shape, **decreased circulating antibody level**, global developmental delay, short foot | 17 years |
| 314461 | female | 4:102152033-104580942 | 2.43 Mb | De novo / Heterozygous | Delayed gross motor development, short stature | 1 year |
| 384471 | male | 4:102103901-103020291 | 916 Kb | Unknown / Heterozygous | Global developmental delay | 4 years |
| 400942 | female | 4:101921665-105168081 | 3.25 Mb | De novo / Heterozygous | Abnormal palate morphology, abnormality of the outer ear, abnormality of toe, arachnodactyly, delayed speech and language development, fine hair, finger clinodactyly, joint laxity, long eyelashes, microdontia, narrow mouth, nasal speech, pes planus, postaxial hand polydactyly, short palpebral fissure, spotty hyperpigmentation, supernumerary tooth, wide nasal base, wide nasal bridge | 4 years |
| 402229 | male | 4:92221672-106721239 | 14.50 Mb | Imbalance arising from a balanced parental rearrangement / Heterozygous | Abnormality of the cardiovascular system, feeding difficulties in infancy, horseshoe kidney, incoordination, low posterior hairline, pectus carinatum, ptosis | 19 years |

| Patient ID | Sex | Chr 4 deletion (coordinates, GRCh38) | Size | Inheritance / Genotype | Phenotype (s) | Age at last clinical assessment |
|---|---|---|---|---|---|---|
| 415252 | male | 4:96898260-104297409 | 7.40 Mb | Unknown / Heterozygous | n.a. | 4 years |
