# Supplementary material for: Common Variable Immunodeficiency and Neurodevelopmental Delay Due to a 13Mb Deletion on Chromosome 4 Including the NFKB1 Gene: A Case Report
Source: Front Immunol. 2022 Jun 17;13:897975. doi: 10.3389/fimmu.2022.897975 (PMC9247144; doi:10.3389/fimmu.2022.897975)
Supplement: Supplementary file 2 [file DataSheet_2.pdf]

Common variable immunodeficiency and neurodevelopmental delay due to a 13Mb deletion on chromosome 4 including the *NFKB1* gene: a case report

Clara Franco-Jarava, Irene Valenzuela, Jacques G. Riviere, Marina Garcia-Prat, Mónica Martínez-Gallo, Romina Dieli-Crimi, Neus Castells, Laura Batlle-Masó, Pere Soler-Palacin, Roger Colobran

Supplementary methods

Microarray-based comparative genomic hybridization (aCGH)

DNA copy-number variations were investigated using an 8x60K CytoSure Constitutional v3 array (OGT, product number: 020045) according to manufacturer recommendations on DNA extracted from peripheral blood with GENTRA Puregene Kit (Qiagen, Hilden, Germany). Data analysis was performed by Cytogenomics 2.1. software with ADM-2 algorithm and a minimum of 3 consecutive probes to detect an anomaly. Reference genome: GRCh37 (hg19).

Western Blot

Cell isolation

Peripheral blood was collected from the patient and a healthy donor in sodium heparin collection tubes. Peripheral blood mononuclear cells (PBMCs) were isolated via Lymphoprep™ density medium (Stemcell technologies), manually counted using an inverted microscope, with cell viability >90% (assessed by Trypan Blue exclusion), and suspended in completed RPMI 1640 medium (with fetal bovine serum, glutamin, penicillin, streptomycin and HEPES buffer) at a concentration of $1x10^6$ cells/ml.

Cell stimulation

$1x10^6$ PBMCs were seeded in 24-well plates. PBMCs were then cultured at 37°C, 5% CO_2 in completed RPMI 1640 medium alone, or stimulated with 0,1 µg/mL phorbol 12-myristate 13-acetate (PMA) (Sigma Aldrich) + 2 µg/mL ionomycin (Merck) for 0, 30 and 60 minutes.

Western Blotting

PBMC pellets were resuspended in 150 µL/$5x10^6$ cells of Pierce™ RIPA lysis buffer (Thermo Scientific) with Halt^{TM} Protease and Phosphatase Inhibitor Cocktail (Thermo Scientific). Total protein content was estimated by Pierce™ BCA Protein Assay Kit (Thermo Scientific) following the manufacturer's protocol. A total of 22.5 µg of protein were loaded onto a 4-20% CriterionTM TGXTM Precast Gel (Bio-Rad). The separated proteins by electrophoresis were transferred onto PVDF membrane through a Trans-BlotR Turbo^{TM} Transfer Pack (Bio-Rad), and blocked with 5% skim milk in TBS-T for 1.5 h with agitation at room temperature. Membranes were incubated with primary antibodies (see table below) overnight at 4ºC. Membranes were then washed in TBS-T and incubated with horseradish peroxidase (HRP)-conjugated goat anti-

rabbit and HRP-conjugated goat anti-mouse antibodies for 1 h at room temperature (see table below). The membranes were washed and visualized with SuperSignal™ West Pico PLUS Chemiluminescent Substrate (Thermo Scientific) using an Odyssey® XF Imaging System (Li-Cor).

List of antibodies used:

**Target**	**Brand, reference**	**Host**	**Dilution**
P-NFkB p105 (Ser933)	Cell signaling, 4806S	Rabbit	1:1000
NFkB p105/p50	Cell signaling, 3035S	Rabbit	1:1000
β-Actin	Thermo Scientific, MA5-15739	Mouse	1:5000
Anti-rabbit IgG, HRP	Cell signaling, 7074S	Goat	1:6000
Anti-mouse IgG, HRP	Thermo Fisher Scientific	Goat	1:20000
